# Protocol for open-label randomized clinical trial of intensive surveillance *versus* standard postoperative follow-up in patients undergoing surgical resection for oesophageal and gastric cancer

**DOI:** 10.1093/bjs/znad180

**Published:** 2023-06-28

**Authors:** Sheraz R Markar, Alice Guazzelli, Amy Taylor, Laura Lloyd Jones, Susan Dutton, Vidoushee Jogarah, Clare Brittain, Nick Maynard, David Cromwell, Filipa Landeiro, Tim Underwood, Jesper Lagergren, Fergus Gleeson, Alan Moss, Tom Crosby

**Affiliations:** Nuffield Department of Surgery, University of Oxford, Oxford, UK; Department of Oesophago-Gastric Surgery, Oxford University Hospitals NHS Foundation Trust, Oxford, UK; Nuffield Department of Surgery, University of Oxford, Oxford, UK; Nuffield Department of Surgery, University of Oxford, Oxford, UK; Nuffield Department of Surgery, University of Oxford, Oxford, UK; Oxford Clinical Trials Research Unit, University of Oxford, Oxford, UK; Oxford Clinical Trials Research Unit, University of Oxford, Oxford, UK; Oxford Clinical Trials Research Unit, University of Oxford, Oxford, UK; Nuffield Department of Surgery, University of Oxford, Oxford, UK; Department of Oesophago-Gastric Surgery, Oxford University Hospitals NHS Foundation Trust, Oxford, UK; Clinical Effectiveness Unit, Royal College of Surgeons of England, London School of Hygiene and Tropical Medicine, London, UK; Health Economics Research Centre, University of Oxford, Oxford, UK; Department of Surgery, University of Southampton, Southampton, UK; Department of Molecular Medicine, Karolinska Institutet, Stockholm, Sweden; Department of Surgery, Kings College London, London, UK; Department of Radiology, Oxford University Hospitals NHS Trust, Oxford, UK; Action Against Heartburn, Patient Association, London, UK; Department of Oncology, Cardiff and Vale University Health Board, Cardiff, UK

Despite recent improvements in oncological and surgical treatment for patients with oesophageal and gastric cancer, 60 per cent of those with locally advanced disease who are treated with curative intent will develop tumour recurrence and die within 3 years of completing treatment. In the absence of robust scientific evidence, national or international guidelines have failed to reach consensus on the optimal surveillance strategy after primary treatment of oesophageal or gastric cancer.

The primary research question of the proposed RCT is: does the routine use of a structured follow-up programme with regular radiological and endoscopic investigations improve survival in patients who have had surgical treatment for oesophageal or gastric cancer with curative intent?

The aim is to assess whether structured follow-up, including radiological and endoscopic investigations, after completion of treatment with curative intent improves survival in patients with oesophageal or gastric cancer. The secondary aims are to determine the impact of structured post-treatment surveillance on the detection and treatment of cancer recurrence and health-related quality of life, including anxiety, and to assess the cost-effectiveness of routine clinical, radiological, and endoscopic investigations compared with the current practice.

A national, prospective, multicentre RCT of structured follow-up, including radiological and endoscopic investigations, *versus* standard clinical follow-up will be undertaken. The setting will be at least 24 large oesophagogastric cancer centres in the UK. The aim is to recruit 952 patients receiving surgical resection for curatively intended treatment of oesophageal or gastric cancer with or without neoadjuvant/adjuvant chemo(radio or immuno)therapy. At 4–12 weeks after surgery for oesophageal or gastric cancer, patients will be assessed for eligibility for inclusion in the trial. Patients will be randomized 1 : 1 to receive either intensive follow-up for up to 3 years, with clinical and CT investigation every 6 months and endoscopy at 12 months, or to current standard National Health Service follow-up, which comprises clinical review at 6 and 12 months followed by targeted investigation as required based on the onset of new symptoms. The primary outcome is 3-year all-cause mortality and secondary outcomes include health-related quality of life (including anxiety), 3-year disease-specific mortality, pattern and treatment of tumour recurrence, and cost-effectiveness of follow-up in both study arms. Patients will be followed up either in clinic or via telephone at baseline, and 6, 12, 18, 24, 30, and 36 months after randomization.

The total duration of the trial is 80 months. Recruitment will last 32 months and there will be a formal stop/go review of the internal pilot in month 15 of the project (9th month of recruitment) to ensure that, at a minimum, 9 centres are active and recruiting at least 2 patients per centre per month. Data from patients in the internal pilot phase will be included in the final analysis.

It is anticipated that the study results will change national guidelines for oesophageal and gastric cancer and thus affect over 4000 patients who undergo surgery in the UK annually. The findings of this RCT will inform national and international guidelines for patients with oesophageal and gastric cancer, as this will be the first trial to provide robust evidence concerning the value of surveillance in this population.

## Data Availability

There are no new data in this manuscript.

